# Design of a Planner-Based Intervention to Facilitate Diet Behaviour Change in Type 2 Diabetes

**DOI:** 10.3390/s22072795

**Published:** 2022-04-06

**Authors:** Kevin A. Cradock, Leo R. Quinlan, Francis M. Finucane, Heather L. Gainforth, Kathleen A. Martin Ginis, Elizabeth B.-N. Sanders, Gearóid ÓLaighin

**Affiliations:** 1 Physiology Department, School of Medicine, National University of Ireland Galway, H91 TK33 Galway, Ireland; k.cradock1@nuigalway.ie (K.A.C.); leo.quinlan@nuigalway.ie (L.R.Q.); 2 Electrical & Electronic Engineering, School of Engineering, National University of Ireland Galway, H91 TK33 Galway, Ireland; 3 Department of Health & Nutrition Science, Atlantic Technological University, Ash Lane, F91 YW50 Sligo, Ireland; 4 CÚRAM SFI Research Centre for Medical Devices, National University of Ireland Galway, H91 TK33 Galway, Ireland; francis.finucane@nuigalway.ie; 5 Bariatric Medicine Service, Centre for Endocrinology, Diabetes and Metabolism, Health Research Board Clinical Research Facility, Galway University Hospitals, H91 TK33 Galway, Ireland; 6 School of Health and Exercise Sciences, Faculty of Health and Social Development, The University of British Columbia, Kelowna, BC V1V 1V7, Canada; heather.gainforth@ubc.ca (H.L.G.); kathleen_martin.ginis@ubc.ca (K.A.M.G.); 7 Department of Medicine, University of British Columbia, Vancouver, BC V6T 1Z3, Canada; 8 Centre for Chronic Disease Prevention and Management, Faculty of Medicine, University of British Columbia, Kelowna, BC V1V 1V7, Canada; 9 International Collaboration on Repair Discoveries, University of British Columbia, Vancouver, BC V6T 1Z3, Canada; 10 Department of Design, The Ohio State University, 100 Hayes Hall, 108 North Oval Mall, Columbus, OH 43210, USA; liz@maketools.com

**Keywords:** diet behaviour change, type 2 diabetes, diet intervention, COM-B model, behaviour change theory, behaviour change techniques, human-centred design

## Abstract

Diet behaviour is influenced by the interplay of the physical and social environment as well as macro-level and individual factors. In this study, we focus on diet behaviour at an individual level and describe the design of a behaviour change artefact to support diet behaviour change in persons with type 2 diabetes. This artefact was designed using a human-centred design methodology and the Behaviour Change Wheel framework. The designed artefact sought to support diet behaviour change through the addition of healthy foods and the reduction or removal of unhealthy foods over a 12-week period. These targeted behaviours were supported by the enabling behaviours of water consumption and mindfulness practice. The artefact created was a behaviour change planner in calendar format, that incorporated behaviour change techniques and which focused on changing diet behaviour gradually over the 12-week period. The behaviour change planner forms part of a behaviour change intervention which also includes a preparatory workbook exercise and one-to-one action planning sessions and can be customised for each participant.

## 1. Introduction

Type 2 diabetes is one of the fastest-growing diseases worldwide, affecting 9% of the world’s population in 2019 [1]. Type 2 diabetes and obesity are closely linked and a recent study from the U.S. reporting obesity prevalence rates highlighted that nearly one in two adults will have obesity and one in four will have severe obesity by 2030 [2]. Type 2 diabetes is largely attributed to excess body weight resulting from sedentary lifestyles and unhealthy dietary behaviours [3]. 

Diet behaviour is extremely complex and is influenced by the interplay of individual factors, social and physical environments and macro level environments [4] in addition to neuroendocrine and genetic influences [5]. Changing diet behaviour is challenging and requires a sophisticated approach to understanding and implementing appropriate techniques to support behaviour change. A recent study by our research group used a design probe methodology to identify barriers and facilitators associated with adopting and maintaining healthy diet and physical activity behaviours in persons with type 2 diabetes [6]. That study identified the food environment, mental health, work schedule, planning, social support, diet cravings, economic circumstances and energy as barriers to diet behaviour change, with planning, food environment, social support and economic circumstances also identified as facilitators [6]. Overcoming these barriers and taking advantage of these facilitators to changing diet behaviour can be supported through the application of appropriate behaviour change techniques (BCTs) [7].

**Figure 1:**
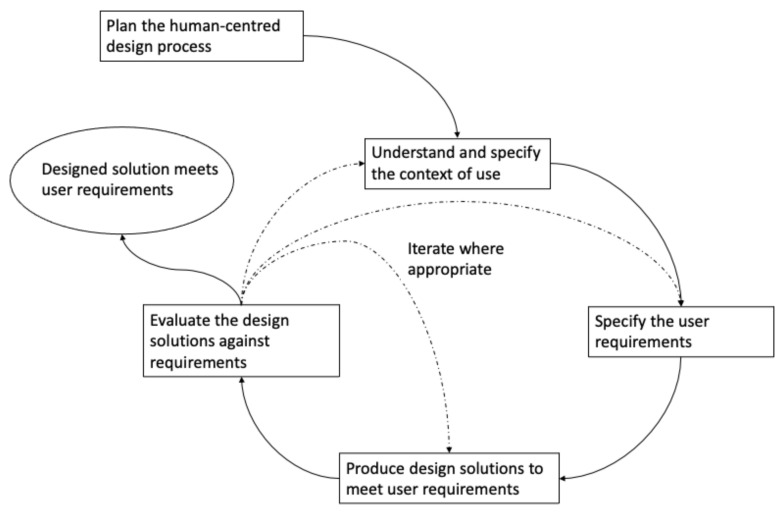
Human-centred design process based on ISO 9241-210 Ergonomics of human–system interaction—Part 210: Human-centred design for interactive systems. The solid lines represent transitions that must occur and the dotted lines are transitions that may occur depending on how the processes evolve.

In addition to basing interventions on a theoretical framework and the inclusion of relevant BCTs, a congruent approach is required to understand how best to apply these models and techniques to specific intervention contexts and individuals in the most effective way [8]. One way to achieve this is through a human-centred design (HCD) approach, which places the person using the design outcome at the centre of the design process [9]. Key elements of the HCD process include (i) having an explicit understanding of users, tasks and environments; (ii) users are involved throughout the design process; (iii) the design is refined by and progressed through user evaluation; (iv) the design is carried out in an iterative manner; (v) the design addresses the entire user experience and (vi) the design is facilitated by a multi-disciplinary design team [10] (Figure 1). 

Our objective was to design an artefact, using an HCD process, to support participants with type 2 diabetes to reduce their consumption of unhealthy food and increase their consumption of healthy food. The Behaviour Change Wheel framework of Michie et al. provided a well-established framework for the design of behaviour change interventions [11] and has been applied to the design of a wide range of interventions, including diet interventions [12,13]. The aim of this study was to design a customised “artefact” to enhance diet behaviour in type 2 diabetes using a human-centred design methodology. The specific aims of this paper are to describe the rationale for and the design of an artefact designed to support diet behaviour change in persons with type 2 diabetes.

## 2. Materials and Methods

### 2.1. User Research

**Figure 2:**
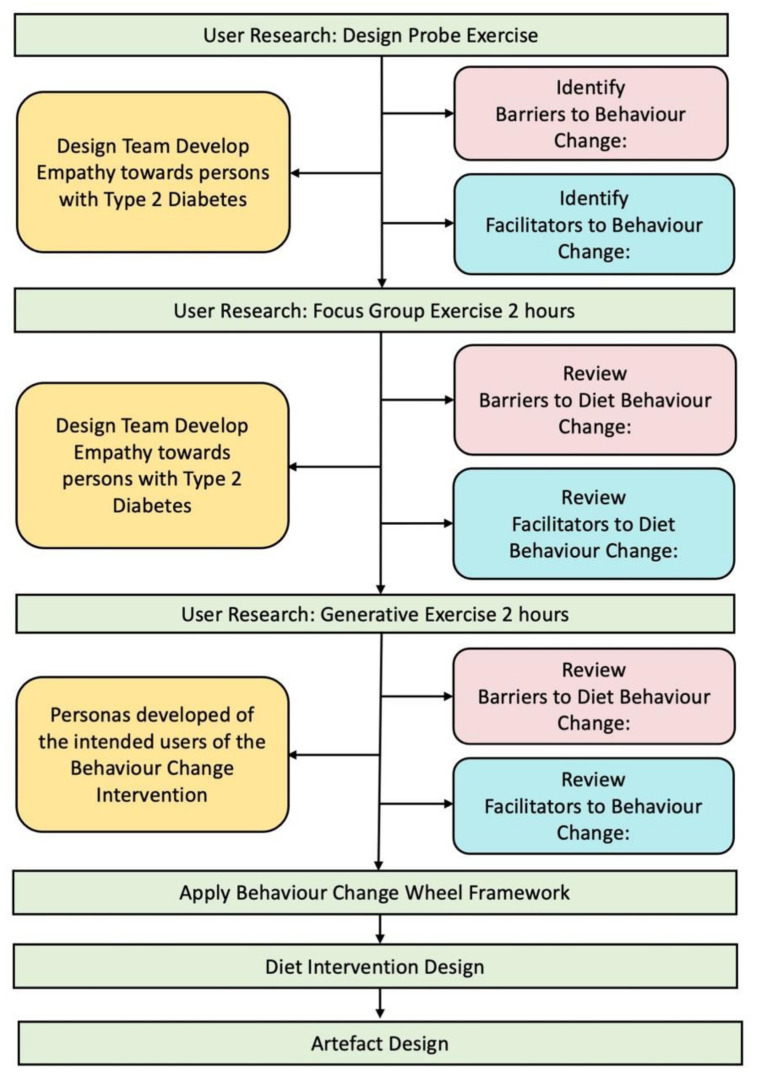
Summary of the artefact design process.

In designing an artefact to support diet behaviour change in type 2 diabetes, we adopted a human-centred design (HCD) methodology [9]. In human-centred design, the start of the design process is user research, where we seek to better understand the needs of the user and to better understand the context in which the proposed artefact will be used. User research was the first step in the artefact design process and was carried out using a design probe exercise, a focus group exercise and a generative exercise. The process adopted is summarised in Figure 2. The second step in the artefact design process was to apply the Behavioural Change Wheel framework using the data collected through user research to establish the key features of the required behaviour change intervention. The third step in the design process was to implement the design of the intervention to incorporate the features of the behaviour change intervention. The fourth step was to design the artefact to deliver the proposed intervention. 

#### 2.1.1. Design Probe Exercise

The first user research process used was the design probe exercise [6], where a design probe was completed by eighteen type 2 diabetes (T2D) participants over 31 days. The design and implementation of this probe was described in detail in Cradock et al. [6].

#### 2.1.2. Focus Group Exercise

The second user research process used was a focus group exercise, carried out with a subset of the participants who had completed the design probe exercise, centred on a discussion on the barriers and facilitators to diet behaviour change. The focus group sessions were carried out at NUI Galway in small groups of 3–4 participants for ~2 h, with two members of the research team combining roles of moderator, audio recorder and time-keeper. The focus groups began with a short period of reflection, where participants read through their completed design probe, followed by an interactive, informal presentation and group discussion of summarised data of the identified barriers and facilitators, relating to the adoption and maintenance of healthy diet behaviours.

#### 2.1.3. Generative Exercise

**Figure 3:**
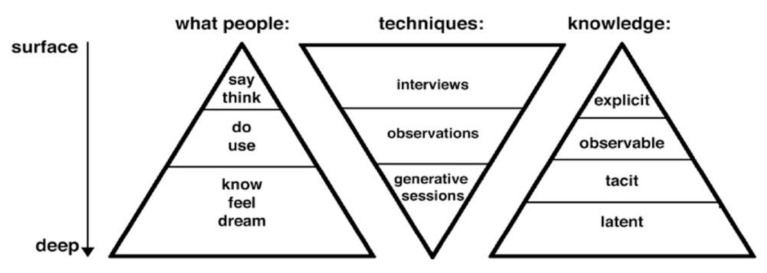
Levels of knowledge achieved with different user research techniques [14].

The third user research process used was a generative exercise, which centred on the participants’ ideas for changing diet behaviours. Conventional user research techniques, such as interviews, observations and focus groups, uncover explicit and observable knowledge about contexts [14]. According to Sleeswijk Visser et al. [14], the main limitation of these conventional techniques for designers of future products is that they offer a view on people’s current and past experiences, but provide little data about the future. To learn about potential future experiences, we need to explore people’s hopes, dreams, fears, aspirations and ideas [14]. Sanders describes how these generative techniques provide a mechanism to reveal future states of people and can reveal tacit knowledge and expose latent needs (Figure 3). What people experience is often determined by *tacit* knowledge or *latent* needs and is often difficult to articulate. With generative techniques, participants are guided in small steps to construct and express deeper levels of knowledge about their experiences. Sleeswijk Visser et al. describe how the use of generative techniques makes it “possible to get access to a hidden world of user experience, and thereby build a better understanding of it, which can then be used for design purposes” (p. 5) [14].

**Figure 4:**
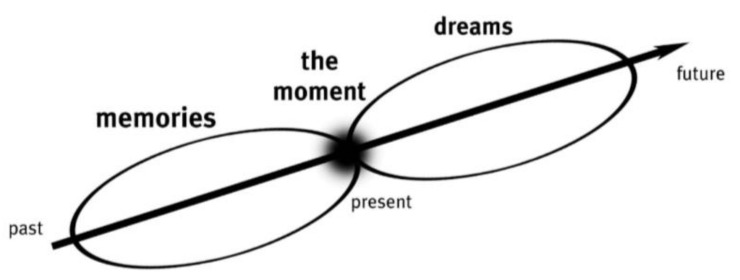
During a generative exercise a ‘hidden world of user experience’ is briefly accessed by the participant in ‘the moment’ where memories and imagination meet [15].

Sanders describes experience as ephemeral, i.e., lasting only a moment [15]. Sanders refers to experiences that have already been lived as *memories* and experiences not yet lived or felt but imagined as *dreams*. Sanders describes experiencing as the point “where memory and imagination meet” (Figure 4) [15] and that the present moment is “inextricably woven into past memories” [15]. Sanders continues that “we interpret what is happening around us with reference to our past experiences” and the present moment “is also tightly coupled to the dreams of our imagination” [15]. Sanders postulated that during a generative exercise, the present experience is interwoven with memories of the past and dreams of the future and that this evoking of people’s dreams will show us how their future could change for the better, in other words, it will reveal latent needs [15].

The generative session process was carried out immediately after the focus group session for an additional ~2 h. Participants were asked to draw, write or create using two-dimensional (2D) or three-dimensional (3D) material, some ‘thing’ that might help them to overcome the barriers and take advantage of the facilitators relating to their diet and physical activity behaviours. The research team worked on their own ideas alongside participants (while not sharing those ideas during the process to avoid bias), which created a positive co-working atmosphere. Participants then individually presented their completed designs and explained in their own words what they had created, these presentations were video recorded. 

### 2.2. Behaviour Change Implementation Framework

**Figure 5:**
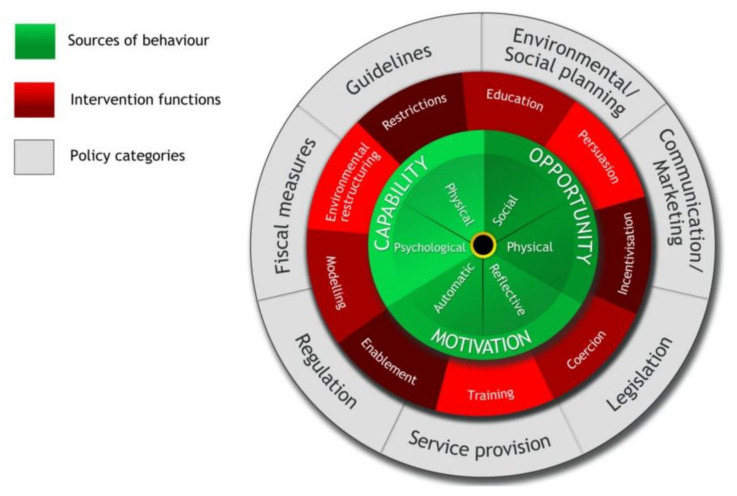
The behaviour change wheel framework [11].

**Figure 6:**
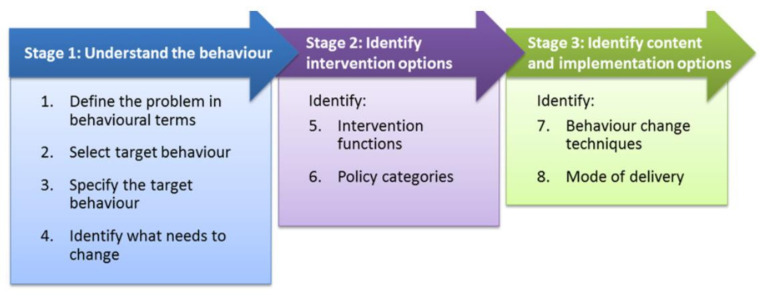
The steps in the Behaviour Change Wheel framework [11].

In designing the artefact to support diet behaviour change in type 2 diabetes, we adopted the Behaviour Change Wheel (BCW) framework for the design of the behaviour change intervention [11]. The BCW framework (Figure 5) of Michie et al. [11] was selected as the framework to design the behaviour change intervention. Embedded in the Behaviour Change Wheel framework is the COM-B model, which proposes that for a behaviour to occur, you must have the physical and psychological capability, the physical and social opportunity and the automatic and reflective motivation to carry out that behaviour [11]. The Behaviour Change Wheel provides a step-by-step framework (Figure 6) for designing a behaviour change intervention and this step-by-step approach was adopted in this study.

The first step in the Behaviour Change Wheel (BCW) framework is to understand the behaviour. This requires that (i) the problem be defined in behavioural terms, (ii) select the target behaviour, (iii) specify the target behaviour and (iv) identify what needs to change.

The second step in the Behaviour Change Wheel framework is to identify intervention options and this involves identifying intervention functions and policy categories (which is not relevant in this case). The third step in the Behaviour Change Wheel framework is to identify content and implementation options and this involves identifying the behaviour change techniques (BCTs) to be used and the mode of delivery of those BCTs.

### 2.3. Intervention Design

The intervention design was informed by the user research process and the outcomes of the Behaviour Change Wheel framework. The BCW intervention functions and user research identified diet barriers and facilitators which informed the actions to be taken in the intervention to address the identified barriers and leverage the identified facilitators.

### 2.4. Artefact Design

The artefact design was informed by the user research process, the outcomes of the BCW framework and the intervention design process, where the required actions were translated into design artefact content. It was proposed at the outset of the design process that the artefact would be paper-based either as an ultimate paper-based artefact or as a paper prototype of a digital artefact in the form of an App.

## 3. Results

### 3.1. User Research

#### 3.1.1. Design Probe Exercise

**Figure 7:**
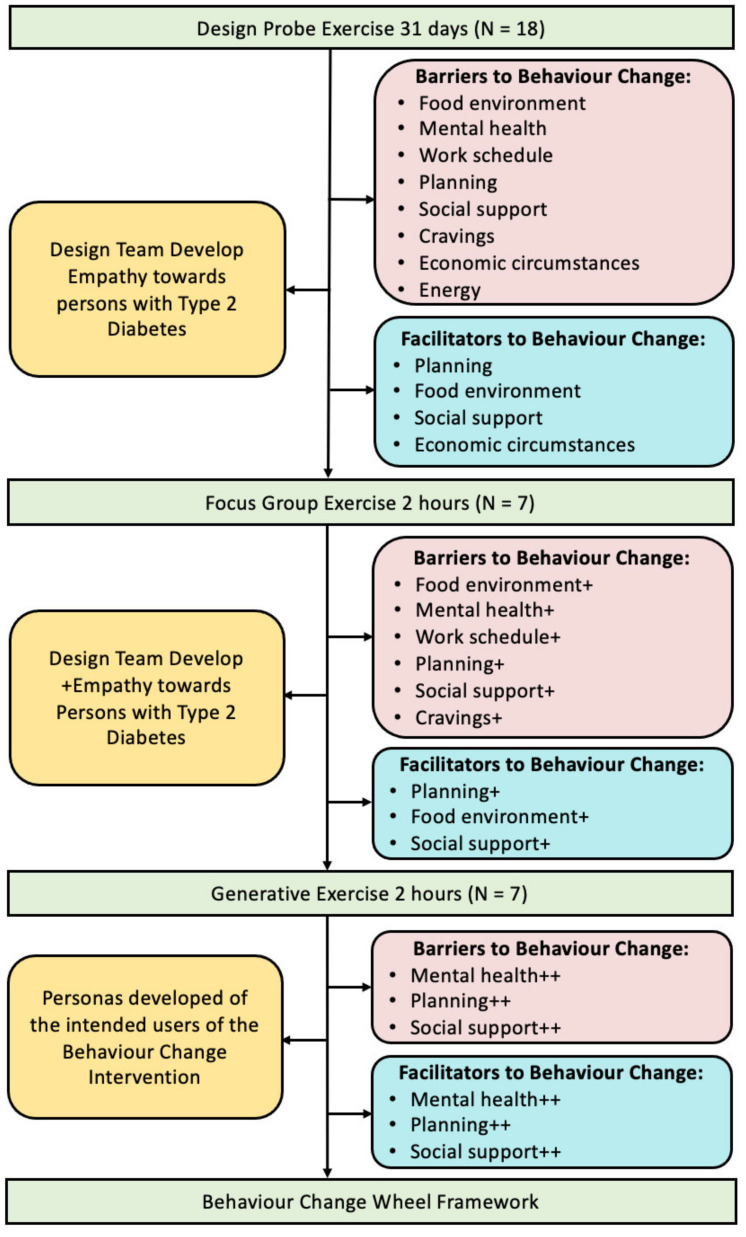
Summary of outcomes of the user research processes. The + denotes which barrier and facilitator themes were further reinforced in the focus group exercises. The ++ denotes which barrier and facilitator themes were again further reinforced in the generative exercises.

This process identified eight themes as barriers (food environment, mental health, work schedule, planning, social support, cravings, economic circumstances, energy) and four themes as facilitators (planning, food environment, social support, economic circumstances) to adopting and maintaining healthy diet behaviours in persons with type 2 diabetes. The process of identifying themes used the thematic analysis process of Braun and Clarke [16] and is described in detail in Cradock et al. [6]. The diet related themes identified in the design probe exercise provided the foundation upon which to build the diet behaviour change intervention. The design probe exercise also served to develop the research team’s empathy towards persons with type 2 diabetes, (Figure 7) as reading the daily content from the participants gave the team real insights into the lived experiences of the participants.

#### 3.1.2. Focus Group Exercise

The focus group exercise helped to reinforce some of the themes from the design probe exercise as barriers (food environment, mental health, work schedule, planning, social support, cravings) and facilitators (planning, food environment, social support). The focus group exercise also served to further develop the research team’s empathy towards persons with type 2 diabetes (Figure 7) as meeting the individuals in person and hearing from them in person, the background to their identified barriers and facilitators, provided the team with greater insight into their lived experiences.

#### 3.1.3. Generative Exercise

The research team found this to be a particularly powerful exercise in that the participants told their ‘story’ visually and then narrated that story in their own words using their imagery to guide the narrative. The participants described their different journeys and struggle with weight loss in the process, highlighting the emotional and psychological complexity that is associated with type 2 diabetes and obesity. This experience assisted in further enhancing the team’s empathy towards the intended end-users. 

Figure 7 summarises the outcomes of the user research processes which were, that the primary barriers and facilitators to diet behaviour change were:

- Planning
  ⚬ Planning and sustaining a plan around diet
  ⚬ Planning to cope with cravings
- Mental health
  ⚬ Low diet self-efficacy
  ⚬ Low self-esteem
  ⚬ Low resilience
  ⚬ Difficulty coping with emotions that might trigger unhealthy eating
- Social support
  ⚬ Influence of family and friends on diet behaviour

During the generative sessions, there was a discussion with the participants on the nature of the proposed design artefact. It was proposed by the research team that a physical paper-based planner be developed with some supporting activities. This was broadly agreed by the participants as being something that they would support as being useful in supporting diet behaviour change.

### 3.2. Human-Centred Design

The human-centred design methodology was adopted throughout the development of the proposed diet intervention, with several iterations of the intervention developed and evaluated with the intended users of the intervention. There were frequent meetings and interactions with the intended users throughout the design process and feedback was sought from them throughout this period to inform the incremental development of the intervention and the artefact. 

One aspect of the HCD process was the decision that the ultimate artefact would be customisable by the user to some extent to meet the unique needs of each user to give the user some sense of ownership of the artefact.

### 3.3. Behaviour Change Wheel Framework

#### Understand the Behaviour

##### Select the Target Behaviour(s)

While the overall goal in T2D is to reduce weight and achieve glycaemic control, the focus for our intervention was diet behaviour, defined as all behaviours relating to increased consumption of healthy food and decreased consumption of unhealthy food. 

**Figure 8:**
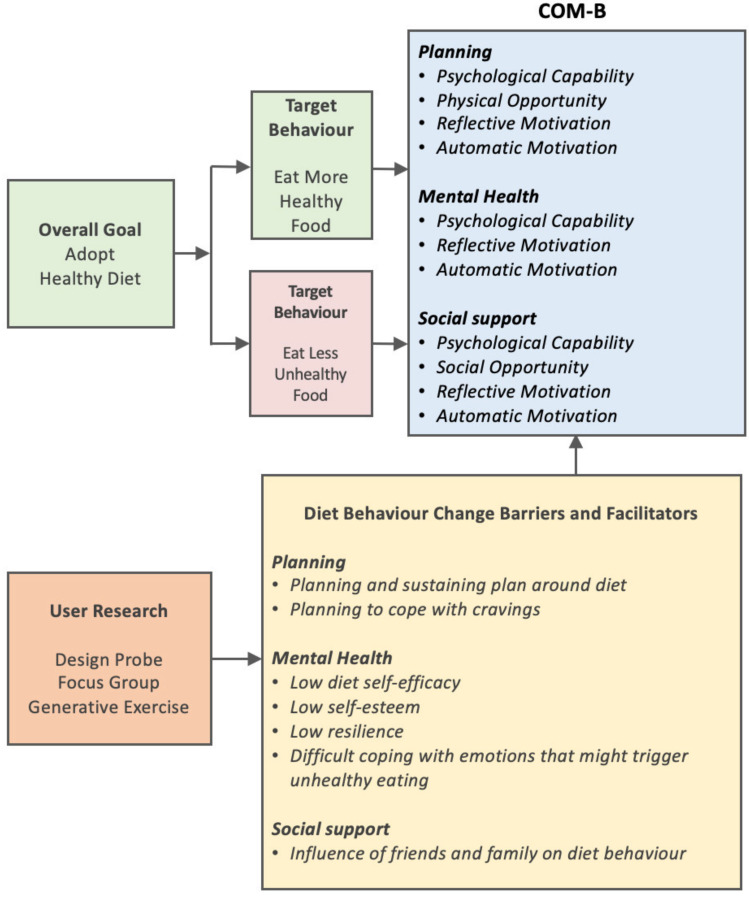
Selecting and specifying the targeted behaviours using the COM-B model.

It was proposed (Figure 8) that diet behaviour change was to be achieved by:

- Adding healthy foods on an incremental basis
- Reducing or removing unhealthy foods on an incremental basis

##### Specify the Target Behaviours

The next step in the behavioural diagnosis involved specifying the targeted behaviours by expressing the barriers and facilitators to perform the targeted behaviour using the COM-B model of capability, opportunity and motivation (Figure 7).

##### Intervention Functions

**Figure 9:**
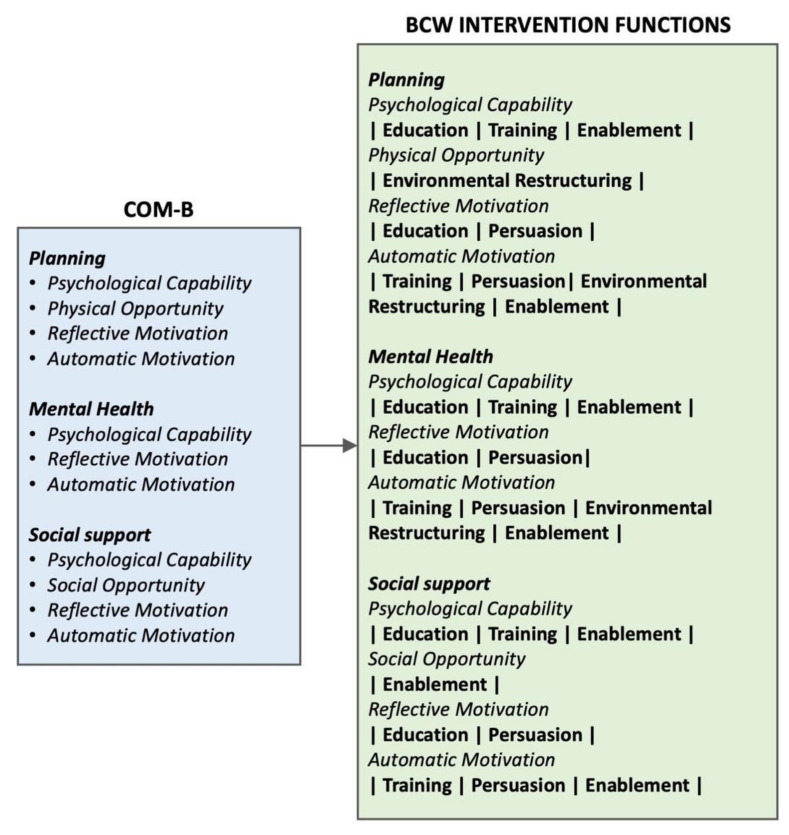
Mapping barriers to intervention functions.

The next step in the process involved identifying the intervention functions to be used to overcome the barriers and facilitators (Figure 9). The intervention functions of ‘coercion’, ‘restriction’ and ‘incentivisation’ were not used as they were considered inappropriate in this setting. The policy categories of the Behaviour Change Wheel were not used as they were not appropriate or feasible for this application. 

### 3.4. Intervention Design

#### 3.4.1. Identifying Behaviour Change Techniques (BCTs) to Be Adopted

Having identified the different BCW intervention functions to be used for the different barrier/facilitator themes, the action to be taken to achieve that intervention was proposed. We described where that action would occur in the diet intervention and what BCTs support these actions, using the numbering scheme from Michie et al.’s taxonomy [17].

Table 1 presents the actions to be taken to address the barriers/facilitators of planning using the intervention functions evolving from the Behaviour Change Wheel framework. Table S1 in the Supplementary Files presents the actions to be taken to address the barriers/facilitators of mental health using the intervention functions evolving from the Behaviour Change wheel framework. Table S2 in the Supplementary Files presents the actions to be taken to address the barriers/facilitators of social support using the intervention functions evolving from the Behaviour Change wheel framework. Table 2 summarises the combined actions for planning, mental health and social support, indicating where in the diet intervention the actions will occur and what BCTs are represented by these actions, using the taxonomy of Michie et al. [17].

Following feedback from participants in the generative sessions, it was proposed that the artefact would be a physical, paper-based planner. To facilitate customisation of this planner for each intervention participant, a preparatory workbook would also be developed for completion by each participant in the proposed intervention, prior to the start of the intervention. Additionally, a preparatory session would take place, where brief action planning and customisation of the planner would be carried out, with the support of the completed preparatory workbook.

We see in Table 2 these three components to the proposed diet intervention:

(i) A preparatory workbook where participants do some preparation work prior to engaging in the core part of the intervention.
(ii) Diet behaviour action plans developed by the participants.
(iii) A planner to support diet behaviour change.

#### 3.4.2. Enablement Intervention Functions

It was proposed that the intervention function of ‘enablement’ which emerged from the BCW framework and which would be used to support diet behaviour change, would be achieved using two enablement functions (i) incorporating a programme of water consumption into the diet intervention and (ii) incorporating daily practice of mindfulness into the diet intervention. 

The daily consumption of water was chosen as an enablement function to facilitate the participants, in a relatively non-challenging way, to increase self-efficacy in a non-diet behaviour. It was also postulated that water practice would act as an enablement function to diet behaviour change as increased water intake has been reported to reduce caloric intake [18] through appetite suppression and reduced intake of/substitution for sugar-based drinks [19]. Additionally, there are significant health benefits associated with increased water intake, as even mild dehydration over a prolonged period is linked to increased risk of hypertension, stroke, coronary heart disease, constipation and urinary tract infections [20]. 

Mindfulness was chosen as an enablement function as it was postulated that mindfulness would help participants to (i) increase their mental resilience, (ii) reduce stress, (iii) reduce anxiety, (iv) help manage depression and (v) help manage low self-esteem and thus indirectly support diet behaviour. Mindfulness has been defined by Kabat-Zinn as “paying attention on purpose, in the present moment, and non-judgementally to the unfolding of experience moment by moment” [21]. Mindfulness has been reported to ameliorate stress [22,23,24], anxiety and depression [23,25,26]. A meta-analytic review of the effects of mindfulness-based therapy (MBT) concluded that MBT improved symptoms of anxiety and depression in multiple disorders and the authors of this study suggested that MBT helped people to respond to stressful situations more reflectively than reflexively [25]. A systematic review of how mindfulness programmes improve mental health and well-being, identified reduced cognitive and emotional reactivity, reduced repeated negative thinking, increased self-compassion and psychological flexibility, as underlying mechanisms of mindfulness-based interventions [27].

In addition to its positive effects on mental health [22,23,24,25,26], practising mindfulness has been demonstrated to have a positive effect on what and how persons eat and drink [28,29,30] and in the management of type 2 diabetes [31]. It has been suggested that mindfulness plays a positive role in different types of problematic eating behaviour, such as restrained eating, emotional eating and external eating [28]. 

**Figure 10:**
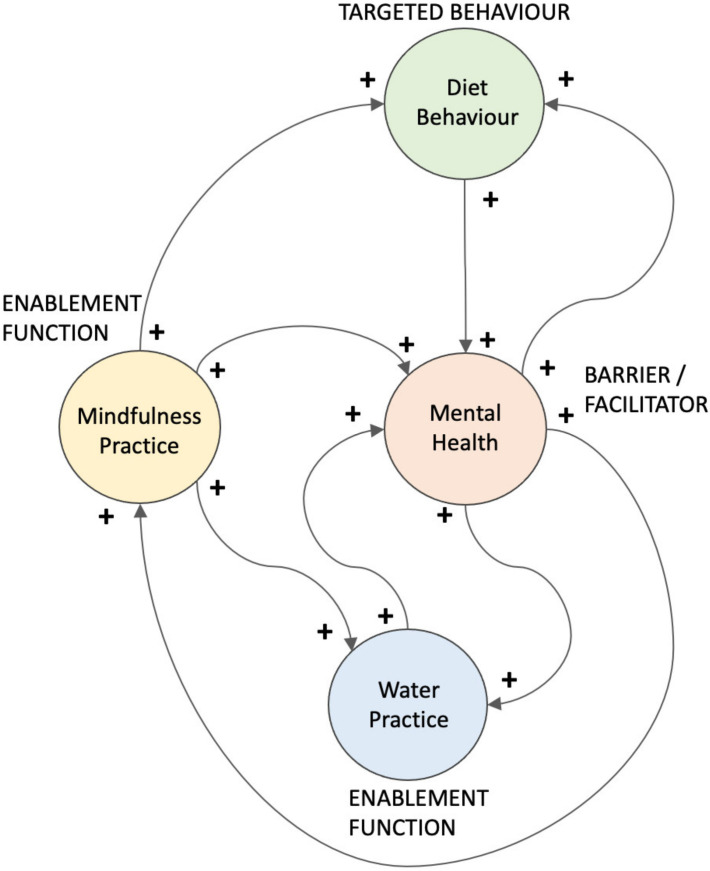
Relationship between the targeted diet behaviour, the barrier/facilitator of mental health and the enablement functions of mindfulness and water practice, showing the presence of multiple self-reinforcing loops.

Figure 10 shows how some elements of the architecture of the diet intervention have the potential to work very effectively together with the presence of self-reinforcing loops. It is postulated that (i) mindfulness practice enhances mental health, water practice and diet behaviour; (ii) mental health enhances mindfulness practice, water practice and diet behaviour; and diet behaviour enhances mental health.

#### 3.4.3. Structure of the Diet Intervention

The proposed overall diet behaviour change programme has a preparatory component and a daily diet practice planning component. The preparatory component involves teaching on barriers, facilitators and action planning exercises to prepare participants for engaging in the daily diet practice. It was proposed by the research team to design the diet intervention as a 12-week programme, which provided sufficient time to gradually introduce diet behaviours on an incremental basis and would not be excessively demanding on the participants. Feedback from participants was that a 12-week programme would be acceptable.

#### 3.4.4. Adding Healthy Food Practices

To support eating more healthy food, a programme of the addition of six incremental healthy food practices over 12 weeks was proposed. These food practices were consistent with the dietary guidance used in routine clinical practice for patients with type 2 diabetes in Ireland [32]. A central part of this guidance is the food pyramid with vegetables and fruit at the bottom of the pyramid suggesting 5–7 servings of each of these per day [33]. The healthy food practice strategy proposed six categories of food (fruit, vegetables I, grains/pulses/beans, nuts/seeds, protein, vegetables II). The vegetable and fruit choices are based on low-GI vegetables and fruits. These categories of food were to be incrementally added. A recent systematic review and meta-analysis highlighted that greater adherence to plant-based diet patterns was linked to a reduced risk of type 2 diabetes and this association was “strengthened when healthy plant-based foods such as fruits, vegetables, whole grains, legumes and nuts, were included in the pattern” [34].

#### 3.4.5. Eliminating or Reducing Un-Healthy Food Practices

To support eating less unhealthy foods, the participants would identify (in the preparatory workbook) unhealthy food practices in their current diet and select five of these practices, that they repeatedly engaged in on a daily basis, that they wanted to reduce or eliminate. The diet practices targeted for reduction or removal were: (i) foods high in sugar [35], fat [36], saturated fats [37] and salt [36]; (ii) foods low in fibre [3]; (iii) highly processed foods [38]; (iv) high glycaemic index foods [3]; and (v) foods containing artificial preservatives or unhealthy additives [39]. These foods have been associated with negative health outcomes in type 2 diabetes prevention or treatment. 

It was proposed that participants would reduce or remove intake of these five foods in their diets on an incremental basis over the 12-week of the programme.

#### 3.4.6. Customisation of the Planner

In line with the HCD approach, while the overall design of the artefact would involve a consistent framework for all participants, it was proposed that the design artefact could be customised to some degree to meet the unique needs of each user. This was to be achieved in the following manner:

- Personalised action plans for diet, mindfulness and water would be developed by each person using brief action planning [40] and incorporated into the customised artefact.
- Inspirational imagery and text would be selected by each person and incorporated into the customised artefact.
- Healthy food practices would be selected by each person from a range provided.
- Unhealthy food removed would be selected by each person based on their diet behaviour.
- Contingency plans were developed by each person in the event of them ‘going off track’ or tempted to ‘go off track’ or ‘have gone off track’ and written up.

The proposed artefact design addressed the identified barriers/facilitators to diet behaviour change in the following manner:

#### 3.4.7. Addressing Planning Challenges

Planning challenges are addressed through (i) the creation of a customised planner which provided daily guidance on actions to be carried out in the diet intervention (Figure 1) and (ii) customised action plans for each food and drink behaviour each week.

#### 3.4.8. Addressing Mental Health Challenges

Mental health challenges are addressed through (i) a comprehensive programme of mindfulness, built up gradually over the 12-week programme; (ii) building self-efficacy through weekly self-belief activities; and (iii) customised weekly motivational quotes and images.

#### 3.4.9. Addressing Social Support Challenges

Social support challenges are addressed by asking the participants to engage in a weekly exercise of identifying themselves as positive role models for family or friends because of the manner in which they are engaging in positive diet behaviour.

### 3.5. Artefact Design

#### 3.5.1. Planner

It was proposed that the artefact would be a physical paper-based planner supported by a preparatory workbook and a preparatory session where brief action planning and customisation of the planner would be carried out. It was also proposed that the planner would be designed according to the following principles:

- The planner programme evolves gradually, adding new behaviours each week.
- Have a highly visually stimulating layout that would inspire the participants to engage with the planner and would evoke positive emotions in the users.
- Would provide the participant with immediate feedback on their weekly progress.
- Would be customised by and for the participant to provide them with a sense of ownership of their planner: (i) weekly inspiration photograph—chosen by the participant, (ii) weekly inspirational quote chosen by the participant and (iii) personalised action plans for proposed changes to behaviours created by the participants themselves with the support of the research team.
- Designed using a human centred design methodology with the design based on comprehensive user research and feedback from users on the design concept.
- The planner would be a physical planner, constructed in the form of a weekly calendar, as a mechanism to explore the feasibility of the approach, prior to its possible implementation in the future as a digital planner.

##### Food Practice

The food practice element of the planner has two components:

- Addition and maintenance of six healthy food practices.
- Reduction in or removal of five unhealthy food practices.

***Adding Healthy Food Practices:***

Starting in week 2, each participant was required to adopt a food practice from one of these six categories (fruit, vegetables I, grains/pulses/beans, nuts/seeds, protein, vegetables II). Each alternate week (2, 4, 6, 8, 10, 12) the participant added a new food practice and maintained that food practice from that category over the remainder of the 12 weeks (See Figure S1, Supplementary Files).

***Eliminating or reducing Unhealthy Food Practices:***

This process aimed to identify (using the ‘Preparatory Workbook’) unhealthy food practices in participants’ diets, and to select five of these practices, that they repeatedly engaged in on a daily basis, that they want to reduce or eliminate. 

##### Mindfulness

The mindfulness component of the planner used the free ‘Smiling Mind’ mindfulness App (https://www.smilingmind.com.au/, accessed on 3 February 2022) which works through two programmes: “An Introduction to Mindfulness” (1 week) and “Mindfulness Foundations’ (6 weeks) followed by a maintenance mindfulness session each day (extended meditation: 20 min) for the remaining five weeks.

##### Water

The water practice goals were set by the research team and gradually increased from drinking 250 mL of water each day for the first week to 1000 mL of water each day by week four, which then continued for the remainder of the 12 weeks. 

#### 3.5.2. Preparatory Workbook

In the ‘Preparatory Workbook’, participants were asked to complete a range of tasks in their own time.

- Record in detail the food they consumed over the last seven days and select five of these food practices which were unhealthy, that they engaged in at least once each day that they would like to remove or reduce over the course of the 12-week programme.
- For the six healthy food behaviours, five unhealthy food behaviours, the mindfulness practice and the water practice participants were asked to:
  - Identify barriers to completing their tasks each day
  - Outline how they were going to overcome these barriers
  - Identify facilitators to completing their tasks each day
  - IIdentify how they could take advantage of these facilitators
  - Identify triggers for going off-track on healthy diet behaviour, coping strategies for previous and future excursions off track

#### 3.5.3. Detailed Diet Intervention Plan

The plan starts with water and mindfulness practice only for week 1 and these practices are added gradually over the 12 weeks of the programme (the diet intervention plan over the 12 weeks is outlined in Figure S1, Supplementary Files). Then on week 2, the first healthy food practice is added where a small quantity of self-selected fruit (from a prescribed selection) is added to the diet with the intention that this food practice will be maintained for the remainder of the 12 weeks of the programme. On week 3, the first reduction/removal of a self-selected unhealthy food is made with the intention that this food practice will be maintained for the remainder of the 12 weeks of the programme. On week 4 the second healthy food practice is added where a small quantity of self-selected vegetables (from a prescribed selection) is added to the diet with the intention that this food practice will be maintained for the remainder of the 12 weeks of the programme. On week 5, the second reduction/removal of a self-selected unhealthy food is made with the intention that this food practice will be maintained for the remainder of the 12 weeks of the programme. On week 6, the third healthy food practice is added where a small quantity of self-selected grains/pulses/beans (from a prescribed selection) is added to the diet with the intention that this food practice will be maintained for the remainder of the 12 weeks of the programme. On week 7, the third reduction/removal of a self-selected unhealthy food is made with the intention that this food practice will be maintained for the remainder of the 12 weeks of the programme. On week 8, the fourth healthy food practice is added where a small quantity of self-selected nuts/seeds (from a prescribed selection) is added to the diet with the intention that this food practice will be maintained for the remaining weeks of the programme. On week 9, the fourth reduction/removal of a self-selected unhealthy food is made with the intention that this food practice will be maintained for the remainder of the 12 weeks of the programme. On week 10, the fifth healthy food practice is added where a small quantity of self-selected meat/fish (from a prescribed selection) is added to the diet with the intention that this food practice will be maintained for the remainder of the 12 weeks of the programme. On week 11, the fifth and last reduction/removal of a self-selected unhealthy food is made with the intention that this food practice will be maintained for the remainder of the 12 weeks of the programme. On week 10, the fifth healthy food practice is added where a small quantity of self-selected vegetables (from a prescribed selection) is added to the diet with the intention that this food practice will be maintained for the remainder of the 12 weeks of the programme.

#### 3.5.4. How the Artefact Design Supports the Diet Intervention

**Figure 11:**
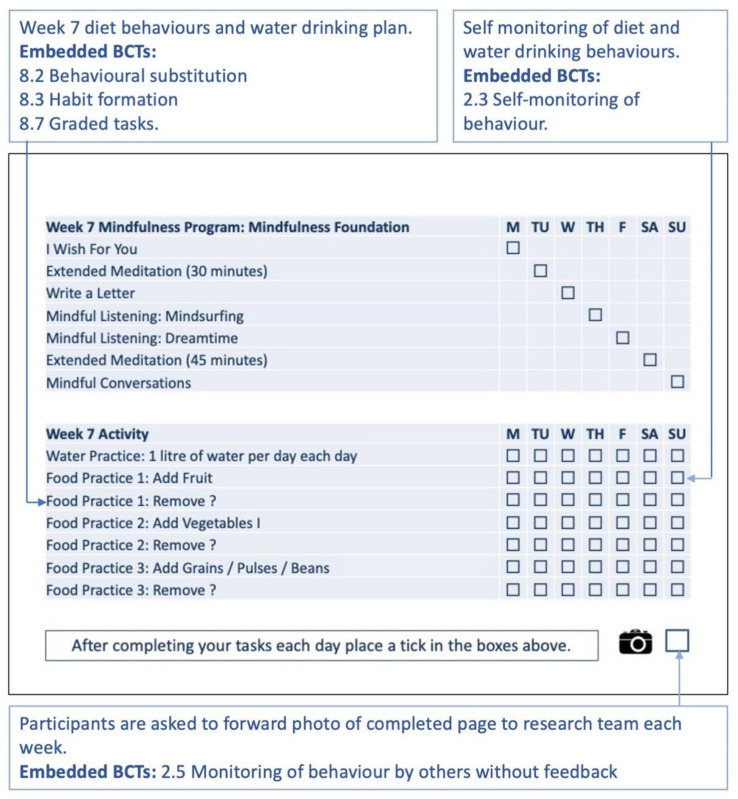
Week 7 monitoring page is part of one A4 page in the planner. The text in the blue boxes is explanatory text and not part of the planner. BCTs used refer to Michie et al. taxonomy [17].

Each week participants are supported by the planner in changing their diet behaviours while engaging in the enablement functions of a daily programme of drinking water and practising mindfulness. The core philosophy of the intervention was to build diet behaviour self-efficacy through self-monitoring, self-reflection and self-praise. A key feature of the lifestyle planner intervention is where participants self-monitor their completion of the planned behavioural tasks each day on their weekly monitoring page, by ticking off tasks once they are completed and at the end of the week, taking a photo of their completed monitoring page for that week and forwarding it to the research team (Figure 11). Participants would be telephoned each week by the research team to monitor their progress and to provide support.

**Figure 12:**
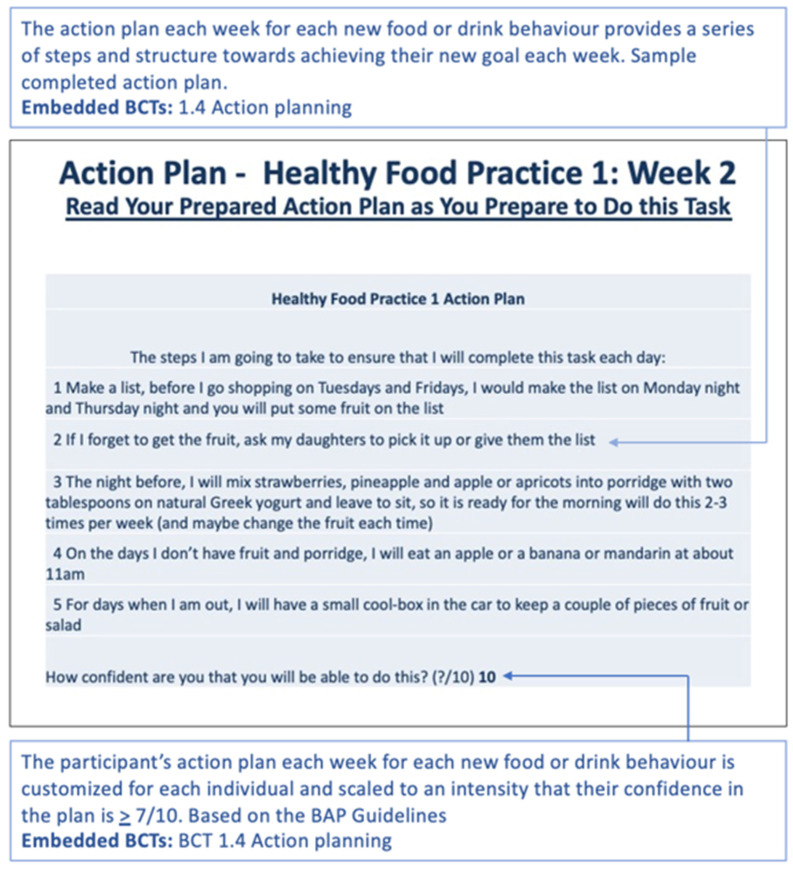
Week 2 developed an action plan for health food practice 1. The text in the blue boxes is explanatory text and not part of the planner. The BCTs used, refer to the Michie et al. taxonomy [17]. The BAP Guidelines, refer to the Gutnick, D. et al. [40].

Individualised action plans for adding healthy foods, removing unhealthy foods mindfulness and water drinking, (developed in the brief action planning sessions) are embedded in the customised planner and presented back to the participant in the relevant part of the planner. The action plan for the new behaviours to be adopted each week are displayed at the start of that week’s section in the lifestyle planner (Figure 12). 

**Figure 13:**
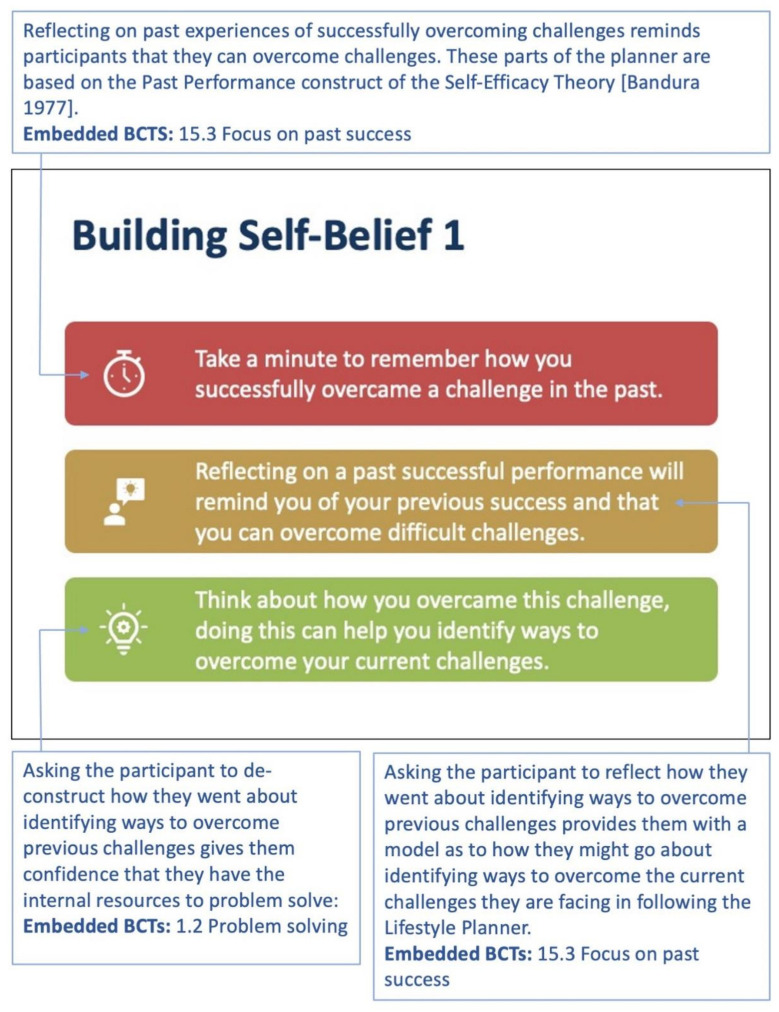
Week 1: On weeks 1, 5 and 9, participants are asked to reflect on past success through a self-reflection process. The text in the blue boxes is explanatory text and not part of the planner. The BCTs used, refer to the Michie et al. taxonomy [17].

A series of statements and associated tasks were embedded in the lifestyle planner each week with a view to developing self-efficacy [41], with one element of self-efficacy targeted each week in the ‘Building Self-Belief’ sections of the planner (Figure 13).

**Figure 14:**
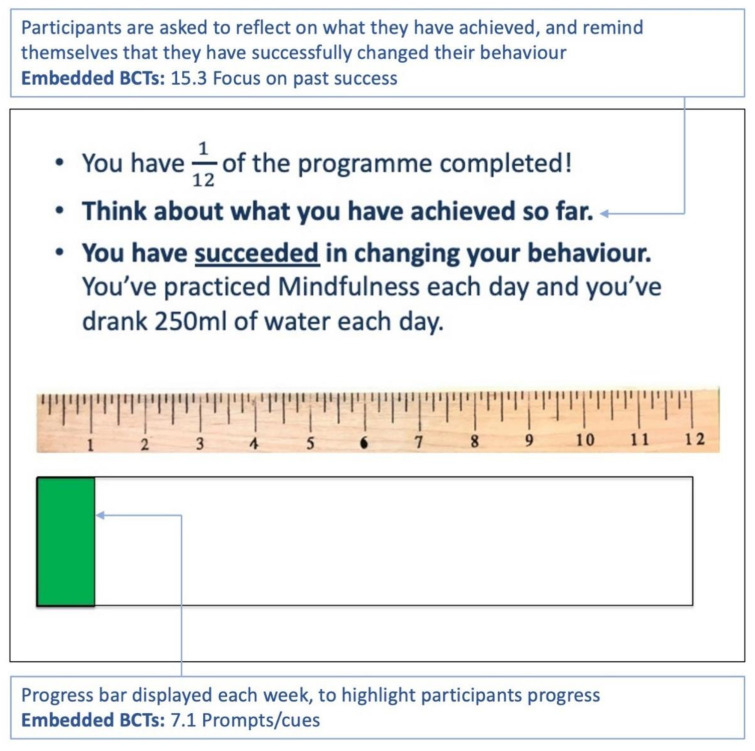
Week 1: The participant is given feedback/encouragement on their progress. The text in the blue boxes is explanatory text and not part of the planner. The BCTs used, refer to the Michie et al. taxonomy [17].

A progress bar at the end of each week illustrates the participant’s progress with the 12-week programme and thus provides feedback on progress (Figure 14). 

**Figure 15:**
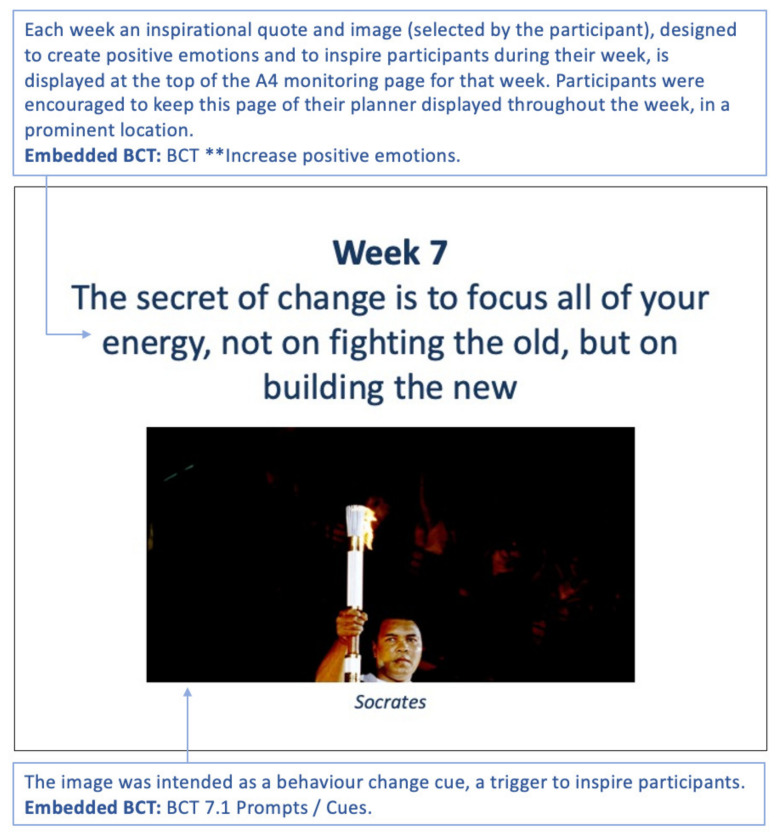
Week 7 inspirational quote and inspirational image selected by the participant. The text in the blue boxes is explanatory text and not part of the planner. BCTs used, refer to the Michie et al. taxonomy [17]. (BCT ** is ‘increase positive emotions’, which is a new BCT scheduled for inclusion in future iterations of the taxonomy.

Each week a customised inspirational quote and motivational image, selected by the user, are displayed on the weekly feedback page (Figure 15). 

**Figure 16:**
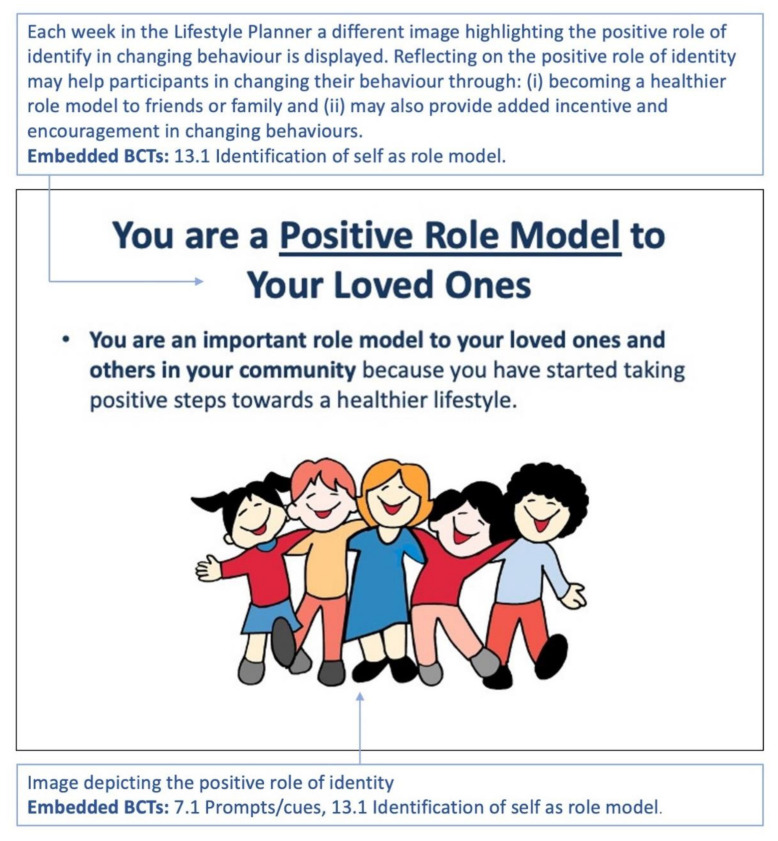
Week 1: A different image and statement depicting the positive role of identity in changing behaviour are shown each week. The text in the blue boxes is explanatory text and not part of the planner. The BCTs used, refer to the Michie et al. taxonomy [17].

The positive role of self-identity in changing behaviour was highlighted each week [17] through the application of the BCT 13.1 ‘Identification of self as role model’ [17], in helping participants identify themselves as a positive role model for family or friends. (Figure 16). 

The planner was developed as a self-standing A4 portrait-oriented planner and was designed so that the weekly monitoring grid (Figure 11) and inspirational image and quote (Figure 15) are on the same page with the inspirational image on top. During use of the planner, the participants would be encouraged to keep the planner (placed at a location of their choosing) open so that the feedback on weekly progress and inspirational imagery and text are visible simultaneously regularly throughout the day.

## 4. Discussion

The aim of this study was to design an ‘artefact’-based intervention to support diet behaviour change in people with type 2 diabetes. Following a human centred design process, incorporating a design probe, focus group and generative exercises and using the Behaviour Change Wheel framework, a diet behaviour change intervention supported by this artefact was designed. The ‘artefact’ was a physical paper-based planner to support diet behaviour change and the proposed intervention also incorporated a preparatory workbook, a brief action planning session to facilitate customisation of the planner for each intervention participant.

A human-centred design (HCD) methodology supported by a multi-disciplinary team was used to design the artefact [9]. The HCD process was a highly immersive process for both the participants and the research team, involving deep engagement with the intended users and requiring considerable time commitment from both parties. 

This deep engagement with the intended users yielded many benefits for the research: (i) supported the research team in better understanding the lives and lived experiences of the proposed ‘artefact’ end-users, (ii) facilitated the development of empathy with the proposed end-users, (iii) helped to create a design based on a deep understanding of the needs of the users and (iv) helped to create an ‘artefact’ customised to meet the unique needs of each end-user. 

The team’s experience with using HCD for this application was very positive and the team would advocate this approach to other researchers designing diet interventions.

The use of HCD for enhancing chronic disease prevention was reviewed by Matheson et al. (2015) and they reported on the positive effect of using the HCD approach in this domain [42]. The HCD process was used by other researchers to enhance mobile nutrition intervention content, by tailoring the intervention content to reflect the context of use and the needs of the full spectrum of users [43]. Another study reported on the use of a HCD approach in a diet app design and the authors recommended its use in the development of future diet-related apps [44]. 

The behaviour change framework adopted was the Behaviour Change Wheel of Michie et al. [11], which is a well-established framework for designing behaviour change interventions and had been previously used in the design of diet interventions [45,46]. 

The BCW framework facilitated a systematic approach to the design of the behaviour change intervention that is grounded in rigorous scientific research. The team’s experience with using the BCW framework for this application was very positive and the team would advocate this approach to other researchers designing diet interventions.

The BCW is cited by several authors as providing a comprehensive and systematic guide to app-based diet behaviour change intervention design, underpinned by relevant evidence and theory [47,48]. The BCW was also successfully adopted in the design of a diet and physical activity intervention in type 2 diabetes [49]. The alignment of the BCW alignment to the COM-B model has also been highlighted as a strength of this approach in intervention design, as the COM-B model encapsulates decades of research on the science of behaviour change [47]. 

The diet intervention aimed to gradually change behaviours by gradually building self-efficacy through the daily enactment of food and drink behaviours and to enhance resilience and emotional regulation through daily mindfulness practice. The process of gradually changing behaviours sought to build potential intervention participants’ diet behaviour self-efficacy, as it has been shown that interventions that focus on strengthening individuals’ beliefs in their ability to control their diet behaviours (self-efficacy) are more successful in both losing weight and maintaining weight loss in the longer term [50]. Bandura concluded that people “integrate diverse sources of information concerning their capability” to carry out a choice behaviour and that they “regulate their choice behaviour and effort expenditure accordingly” [41]. He also suggested that “efficacy expectations” around a choice behaviour “are presumed to influence the level of performance by enhancing intensity and persistence of effort” [41]. Self-efficacy determines if attempts will be made at changing behaviour, in addition to the degree of effort expended when difficulties arise [51]. 

Developing self-efficacy is enabled in the intervention design through starting the process of behaviour change with gradual increases in drinking water, perceived as a less challenging behaviour [52] to begin with, in week one followed by the gradual introduction of a new diet behaviour in week two. Starting the intervention with a less challenging behaviour allows potential intervention participants to experience success early and to gradually build self-efficacy, through the daily enactment of behaviours. The process of the gradual addition of one new healthy diet behaviour on even weeks and the reduction or removal of unhealthy behaviours on odd weeks seeks to further increase self-efficacy, by building success slowly through the gradual adoption of new and maintenance of existing behaviours each week. 

The structure of the action plans, monitoring pages and progress bar in the planner are designed to facilitate the daily enactment of behaviours to gradually build self-efficacy. The building self-belief section also seeks to build self-efficacy through encouraging participants to reflect on previous successful performances, identify role models to emulate, engage social support and remind participants of their capabilities.

The rationale for the inclusion of mindfulness, as a supporting behaviour in the intervention, stemmed from the identified need to enhance participant mental health to support diet behaviour change. Multiple studies have shown that mindfulness enhances mental health by enhancing mental resilience and emotional regulation [22,23,24,53,54] and the role of mindfulness specifically in enhancing diet behaviour has been reported in several studies [28,29,30,31]. 

Different durations of mindfulness-based interventions supporting diet behaviour change have been used in other studies with 11 weeks [55], eight weeks [56] and six weeks [57] reported compared to the 12 weeks in this study. A variety of different methods of delivering the mindfulness interventions have been used, with studies using an app linked to YouTube mindfulness videos [57], workshop presentations and homework tasks [30], an app incorporating audio, video and mindfulness education [55] and text, audio and introductory videos [56]. The focus of mindfulness interventions also varied with certain interventions using an ACT (acceptance commitment therapy) approach [30,56], others using mindfulness education and mindfulness-based stress reduction techniques [55] and other studies focusing on increasing awareness of satiety/hunger cues, self-observation, sensory aspects of eating and mindful movement [57].

An important feature of the intervention design was that the planner to support diet behaviour change is customised to meet some of the unique needs of each intended participant. The research team considered this feature of the artefact’s design to be important in providing the intended participant with a sense of ownership of the artefact, which they would use on a daily basis to support their diet behaviour.

Each individual’s action plan for each diet and supporting behaviour is individually developed by that individual. The other features of the planner that are customised by each participant are weekly motivational quotes and images.

Converting the proposed paper artefact to a smartphone app could potentially facilitate easier peer-support than the paper-based approach, by allowing intervention participants (subject to agreement) to communicate with one another (potentially anonymously) and to offer support and encouragement to one another. An app-based approach would offer additional capabilities, such as structured monitoring of compliance and health outcomes, the opportunity to provide feedback [58], enhanced portability and would potentially facilitate more extensive customisation of the planner. The use of digital approaches to diet behaviour change, such as the use of a digital planning and purchasing platform [59] and digital weight management interventions, with one-to-one health coaching [12], have resulted in sustainable weight loss in obese populations. A systematic review in 2019 concluded that “app-based mobile interventions are effective and highly promising for changing nutrition behaviours and nutrition-related health outcomes” [13]. 

### 4.1. Implications for the Design of Wearable Devices

In this paper, we described in detail, from first principles, the steps to be carried out to design a diet behaviour change intervention using the Behaviour Change Wheel. 

Wearable devices for the management of health and wellness, inevitably require the end-user to change some aspect of their behaviour when engaging with and using the device [60,61]. For example, a fitness tracker requires the user to use the feedback from the tracker to try to increase, for example, the number of steps they complete each day. Thus, a key part of this type of intervention is that the user wears the tracker each day, throughout the day, so that an accurate record of steps is recorded and that the user responds appropriately to reminders and notifications. A digital health-based blood pressure or blood glucose monitoring system requires the user to adhere to a programme of regularly measuring their arterial blood pressure/blood glucose using the system and to use these data in the management of their health. This may seem like an obvious point, but wearable health devices in general, no matter how worthwhile the concept of the wearable health device, are only of benefit to the intended user if they use the device as intended. In other words a wearable device is only of benefit if the intended user engages in the behaviours expected by the designer [62]. However, adherence to wearable health devices can in fact be very poor and the issue of poor compliance to wearable health technology is of particular concern to payers, like insurance companies, who sometimes pay for the wearable technology on the basis of the promised health benefits these devices should provide. However, compliance can be very poor, with the wearable technology unused or used infrequently or its use dropping off over time with no health benefits or reduced health benefits achieved [63]. This is a real challenge for digital healthcare [64].

So how do designers of wearable health devices go about solving this problem? They can start to solve the problem by designing the devices in a manner that will elicit the required behaviour change by the intended user/users, so that the device will be used as intended by its designers. This can be achieved by following the steps we have outlined in this paper:

- Define the problem to be addressed by the wearable device in behavioural terms
- Select the behaviours of the intended user/users of the wearable device that must change for the device to be used as intended
- Specify the behaviours of the intended user/users of the wearable device that must change for the device to be used as intended
- Identify what aspect of these behaviours must change using the COM-B model
- Identify the intervention functions to be used to bring about the required behaviour change using the Behaviour Change Wheel
- Identify the behaviour change techniques (BCTs) to be incorporated into the design of the wearable device to implement these intervention functions
- Identify the mode of delivery of the BCTs in the wearable device

We hope that this paper will provide future designers of wearable health systems with a roadmap to implementing an end-user behaviour change strategy into the design of their digital health system.

The next step in the research described in this paper is to evaluate the proposed diet behaviour change intervention in a pilot study.

#### 4.1.1. Strengths

One of the strengths of this study was the extensive use of the HCD approach, which incorporated a design probe study by this research team on the same population [6]. The rigorous application of the BCW framework and associated COM-B model to behaviour change intervention design is another strength of this study. A large number of BCTs are systematically delivered in the design artefact which supports the proposed intervention. Another strength was the strong multidisciplinary design team that created the intervention design, with psychologists, clinicians, engineers, designers and biological scientists. The feature of the artefact design, where it is customised to some degree to reflect the unique needs and circumstances of each participant, is also a strength of the approach.

#### 4.1.2. Limitations

One of the limitations of this study is the extensive time commitment required by participants. The structure of the paper prototype approach may limit its use to a home or work setting as it does not have the same portability as a smartphone app. Social support was addressed in this planner but not to the same degree as ‘planning’ or ‘mental health’ and should be addressed more comprehensively in future iterations of the intervention, as the importance of the involvement of family members in type 2 diabetes self-management was highlighted in Bennich et al. [65]. 

## 5. Conclusions

A diet behaviour change intervention incorporating a preparatory workbook, one-on-one action planning and a planner was designed by a multidisciplinary team. An HCD approach was used to help design an ‘artefact’ that met the specific needs of its end users. The HCD approach identified barriers/facilitators of ‘planning’, ‘mental health’ and ‘social support’ which provided a platform for the intervention design. The BCW framework provided a behaviour change framework to create a design intervention, which was complemented by the HCD approach. The design philosophy adopted, used a gradual approach to changing diet behaviour. The aim of changing the target behaviours of adding healthy foods and reducing/removing unhealthy foods was supported by the addition of two enabling behaviours: mindfulness practice and water consumption. The planner-based intervention is a novel approach and may provide the framework for a mobile app designed to support diet behaviour change in persons with type 2 diabetes. The next step would be carrying out feasibility testing of the planner-based intervention with type 2 diabetes participants.

## Figures and Tables

**Table 1:** Action to be taken to support the relevant intervention functions associated with the three dimensions of the COM-B model for the barrier/facilitator theme planning.

Planning		
COM-B	Intervention	Description of Action Taken—Direct Mode of Action
Psychological Capability	Education	Teaching on barriers and facilitators to engage in the behaviours. Teaching on action planning for behaviours. Teaching on how to plan through example with the physical planner. Provide feedback on the execution of the plan through the physical planner. Teaching on the different components of self-efficacy.
	Training	Train the participants on planning using the physical planner as an example. Training in self-reflection on the components of self-efficacy.
	Enablement	Programme of mindfulness incorporated into the intervention to support the execution of the behaviours by enhancing psychological capability. Programme of water consumption to provide a means to build self-efficacy in a non-food task.
Physical Opportunity	Environmental Restructuring	Placement of the planner in the home where it is regularly visible to the participant, helps them to view their progress with the intervention.
Reflective Motivation	Education	Teaching on barriers and facilitators to engage in the behaviours. Teaching on action planning for behaviours. Teaching on how to plan through example with the physical planner. Provide feedback on the execution of the plan through the physical planner. Teaching on the different components of self-efficacy. Teaching on the social consequences of behaviour.
	Persuasion	Use inspirational quotes, inspirational images and other celebratory/congratulatory imagery and text throughout the planner to help the participants to reinforce their intensions relating to the intervention as they work through the planner
Automatic Motivation	Training	Repeated use of the planner each day helping to make the behaviours habitual and reducing the likelihood of going off-track. Training on how to avoid going ‘off-track’ or recover if gone ‘off-track’.
	Persuasion	Use inspirational quotes, inspirational images and other celebratory/congratulatory imagery and text to help the participants experience positive emotions about engaging in the behaviour and adhering to the behaviours and help make the behaviours habitual.
	Environmental Restructuring	Placement of the planner in the home where it is regularly visible to the participant as a prompt or a cue to engage in the behaviours.
	Enablement	Programme of mindfulness incorporated into the intervention to support emotional regulation and mental resilience.

**Table 2:** Combined actions to be taken to support the barrier/facilitator themes of planning, mental health and social support, where in the intervention those actions will be taken and the BCT numbers (Table 3) that the action represents are based on Michie et al.’s taxonomy [17]. BCT ** is ‘increase positive emotions’, which is a new BCT scheduled for inclusion in future iterations of the taxonomy.

Combined for Planning|Mental Health|Social Support		
Action Taken	Where	BCT
Teaching on barriers and facilitators to engage in the behaviours.	Preparatory Workbook	1.1, 1.2
Training on how to avoid going ‘off-track’ or recover if gone ‘off-track’.	Preparatory Workbook	1.2, 4.1
Teaching on action planning for behaviours.	Action Planning	1.1, 1.2, 1.4, 1.8, 1.9
Teaching on how to plan through example with the physical planner.	Planner	2.2, 2.3, 7.1
Provide feedback on the execution of the plan through the physical planner.	Planner	2.2, 2.3, 7.1
Teaching on the different components of self-efficacy.	Planner	15.1, 15.3, 16.1
Train the participants on planning using the physical planner as an example.	Planner	2.2, 2.3, 7.1
Training in self-reflection on the components of self-efficacy.	Planner	15.1, 15.3, 16.1
Programme of mindfulness incorporated into the intervention.	Planner App	1.1, 1.4, 2.2, 2.3, 4.1, 8.7, 11.2
Programme of water consumption to provide a means to build self-efficacy in a non-food task.	Planner	1.1, 1.4, 2.2, 2.3, 4.1, 8.7
Placement of the planner in the home where it is regularly visible to the participant.	Planner	7.1, 12.5
Teaching on the social consequences of behaviour.	Planner	5.3, 10.4, 10.5
Repeated use of the planner each day helping to make the behaviours habitual and reducing the likelihood of going off-track.	Planner	2.2, 2.3, 4.1, 7.1, 8.2, 8.3
Use inspirational quotes, inspirational images and other celebratory/congratulatory imagery and text.	Planner	7.1, 10.4, 10.5, 10.9, 13.1, **
Training using repeated exposure to positive emotions elicited using imagery and text in the planner.	Planner	2.2, 2.3, 4.1, 7.1, 8.2, 8.3
Text to prompt participants seek support of family or friends.	Planner Preparatory Workbook	3.1

**Table 3:** BCTs used from Michie’s v1 Taxonomy (BCT ** is ‘increase positive emotions’, which is a new BCT scheduled for inclusion in future iterations of the taxonomy [17]).

BCT No.	BCT
1.1	Goal setting (behaviour)
1.2	Problem solving
1.4	Action planning
1.8	Behavioural contract
1.9	Commitment
2.2	Feedback on behaviour
2.3	Self-monitoring of behaviour
3.1	Social support (unspecified)
4.1	Instruction on how to perform a behaviour
5.3	Information about social and environmental consequences
7.1	Prompts/cues
8.2	Behaviour substitution
8.3	Habit formation
8.7	Graded tasks
10.4	Social reward
10.5	Social incentive
10.9	Self-reward
11.2	Reduce negative emotions
12.5	Adding objects to the environment
13.1	Identification of self as role model
15.1	Verbal persuasion about capability
15.3	Focus on past success
16.3	Vicarious consequences
**	Increase positive emotions

## Data Availability

Not applicable.

## Supplementary Materials

The following are available online at https://www.mdpi.com/article/10.3390/s22072795/s1, Table S1: Action to be taken to support the relevant intervention functions associated with the three dimensions of the COM-B model for the barrier/facilitator theme Mental Health; Table S2: Action to be taken to support the relevant intervention functions associated with the three dimensions of the COM-B model for the barrier/facilitator theme Social Support; Figure S1: Proposed Planner Content over the 12 weeks.
