# Supplementary material for: Design of a Planner-Based Intervention to Facilitate Diet Behaviour Change in Type 2 Diabetes
Source: Sensors (Basel). 2022 Apr 6;22(7):2795. doi: 10.3390/s22072795 (PMC9002653; doi:10.3390/s22072795)
Supplement: Supplementary file 1 [file sensors-22-02795-s001.zip › sensors-1605432-supplementary.pdf]

Table S1. Action to be taken to support the relevant intervention functions associated with the three dimensions of the COM-B model for the barrier/facilitator theme Mental Health

**Mental Health**		
**COM-B**	**Intervention Function**	**Action to be taken**
Psychological Capability	Education	Teaching on the different components of self-efficacy.
	Training	Training in self-reflection on the components of self-efficacy.
	Enablement	Programme of Mindfulness to support the execution of behaviours by enhancing psychological capability.<br>Programme of water consumption to build self-efficacy in non-food task.
Reflective Motivation	Education	Teaching different components of self-efficacy carried out in planner.
	Persuasion	Use inspirational quotes, inspirational images and other celebratory/congratulatory imagery and text throughout the planner to enhance the participants' feelings of self-belief and self-efficacy as they work through the planner.
Automatic Motivation	Training	Training using repeated exposure to positive emotions elicited using imagery and text in the planner.
	Persuasion	Use inspirational quotes, inspirational images and other celebratory/congratulatory imagery and text to help the participants experience positive emotions to enhance feelings of self-belief and self-efficacy.
	Environmental Restructuring	Placement of planner in home where regularly visible to participant as reminder of intervention process and positive emotions associated with it.
	Enablement	Programme of Mindfulness to support emotional regulation and mental resilience.

Table S2. Action to be taken to support the relevant intervention functions associated with the three dimensions of the COM-B model for the barrier/facilitator theme Social Support

Social Support		
**COM-B**	**Intervention Function**	**Action to be taken**
Psychological Capability	Education	Teaching different components of self-efficacy, which can be applied in family and social situation.
	Training	Training in self-reflection on the components of self-efficacy which can be applied in a family and social situation.
	Enablement	Programme of Mindfulness help emotional regulation & mental resilience to manage food behaviours in family and social situations.<br>Prompt participants seek support of family or friends.
Social Opportunity	Enablement	Programme of Mindfulness helps emotional regulation and mental resilience to be able to manage food behaviours in family and social situations.<br>Prompt participants to seek support of family or friends
Reflective Motivation	Education	Teaching on the different components of self-efficacy, which can be applied in a family and social situation.<br>Teaching on the social consequences of behaviour.
	Persuasion	Inspirational quotes, inspirational images and other celebratory/congratulatory imagery and text to enhance the participants' feelings of self-belief and self-efficacy in social and family situations.
Automatic Motivation	Training	Training in self-reflection on the components of self-efficacy can be applied in family and social situations.
	Persuasion	Inspirational quotes, inspirational images and other celebratory/congratulatory imagery and text help participants experience positive emotions to enhance feelings of self-belief and self-efficacy when in family and social situations.
	Enablement	Programme of Mindfulness to support emotional regulation and mental resilience, including in family and social situations.

Week 1: Water Practice 1 **|** Meditation Practice 1-7

Week 2: Water Practice 2 **|** Meditation Practice 8-14 **|** Healthy Food Practice 1: Fruit

Week 3: Water Practice 3 **|** Meditation Practice 15-21 **|** Healthy Food Practice 1: Fruit **|** Remove Unhealthy Food Practice 1

Week 4: Water Practice 4 **|** Meditation Practice 22-28 **|** Healthy Food Practice 1: Fruit **|** Healthy Food Practice 2: Vegetables **|** Remove Unhealthy Food Practice 1

Week 5: Water Practice 3 **|** Meditation Practice 22-28 **|** Healthy Food Practice 1: Fruit **|** Healthy Food Practice 2: Vegetables **|** Remove Unhealthy Food Practice 1 **|** Remove Unhealthy Food Practice 2

Week 6: Water Practice 3 **|** Meditation Practice 29-35 **|** Healthy Food Practice 1: Fruit **|** Healthy Food Practice 2: Vegetables **|** Healthy Food Practice 3: Grains/Pulses/Beans **|** Remove Unhealthy Food Practice 1 **|** Remove Unhealthy Food Practice 2

Week 7: Water Practice 3 **|** Meditation Practice 36-42 **|** Healthy Food Practice 1: Fruit **|** Healthy Food Practice 2: Vegetables **|** Healthy Food Practice 3: Grains/Pulses/Beans **|** Remove Unhealthy Food Practice 1 **|** Remove Unhealthy Food Practice 2 **|** Remove Unhealthy Food Practice 3

Week 8: Water Practice 3 **|** Meditation Practice 23 **|** Healthy Food Practice 1: Fruit **|** Healthy Food Practice 2: Vegetables **|** Healthy Food Practice 3: Grains/Pulses/Beans **|** Healthy Food Practice 4: Nuts/Seeds **|** Remove Unhealthy Food Practice 1 **|** Remove Unhealthy Food Practice 2 **|** Remove Unhealthy Food Practice 3

Week 9: Water Practice 3 **|** Meditation Practice 23 **|** Healthy Food Practice 1: Fruit **|** Healthy Food Practice 2: Vegetables **|** Healthy Food Practice 3: Grains/Pulses/Beans **|** Healthy Food Practice 4: Nuts/Seeds **|** Remove Unhealthy Food Practice 1 **|** Remove Unhealthy Food Practice 2 **|** Remove Unhealthy Food Practice 3 **|** Remove Unhealthy Food Practice 4

Week 10: Water Practice 3 **|** Meditation Practice 23 **|** Healthy Food Practice 1: Fruit **|** Healthy Food Practice 2: Vegetables **|** Healthy Food Practice 3: Grains/Pulses/Beans **|** Healthy Food Practice 4: Nuts/Seeds **|** Healthy Food Practice 5: Meat/Fish **|** Remove Unhealthy Food Practice 1 **|** Remove Unhealthy Food Practice 2 **|** Remove Unhealthy Food Practice 3 **|** Remove Unhealthy Food Practice 4

Week 11: Water Practice 3 **|** Meditation Practice 23 **|** Healthy Food Practice 1: Fruit **|** Healthy Food Practice 2: Vegetables **|** Healthy Food Practice 3: Grains/Pulses/Beans **|** Healthy Food Practice 4: Nuts/Seeds **|** Healthy Food Practice 5: Meat/Fish **|** Remove Unhealthy Food Practice 1 **|** Remove Unhealthy Food Practice 2 **|** Remove Unhealthy Food Practice 3 **|** Remove Unhealthy Food Practice 4 **|** Remove Unhealthy Food Practice 5

Week 12: Water Practice 3 **|** Meditation Practice 23 **|** Healthy Food Practice 1: Fruit **|** Healthy Food Practice 2: Vegetables I **|** Healthy Food Practice 3: Grains/Pulses/Beans **|** Healthy Food Practice 4: Nuts/Seeds **|** Healthy Food Practice 5: Meat/Fish Seeds **|** Healthy Food Practice 6: Vegetables II**|** Remove Unhealthy Food Practice 1 **|** Remove Unhealthy Food Practice 2 **|** Remove Unhealthy Food Practice 3 **|** Remove Unhealthy Food Practice 4 **|** Remove Unhealthy Food Practice 4 **|** Remove Unhealthy Food Practice 5

Figure S1. Proposed Planner Content over the 12 weeks.
